# Mental fatigue in football: methodology and experimental protocol

**DOI:** 10.3389/fspor.2026.1788854

**Published:** 2026-03-25

**Authors:** Diogo Aveiro, Fernando Martins, Mário Pereira, Rodrigo Coimbra, Rodrigo José Mendes, João Pinto, John Kiely, Francisco Campos

**Affiliations:** 1 Department of Physical Education and Sport Sciences, University of Limerick, Limerick, Ireland; 2 Polytechnic University of Coimbra, Coimbra, Portugal; 3 SPRINT–Sport Physical activity and health Research & INnovation cenTer, Polytechnic University of Coimbra, Coimbra, Portugal; 4 Instituto de Telecomunicações—Delegação da Covilhã, Covilhã, Portugal; 5 iNED–Centre for Research & Innovation in Education, Polytechnic University of Coimbra, Coimbra, Portugal

**Keywords:** cognitive load, EEG, ETS, football, mental fatigue, motor performance

## Abstract

Mental fatigue, induced by prolonged cognitive demands, impairs attention, decision-making, and motor coordination, potentially compromising performance in cognitively demanding sports such as football. This study presents a randomised controlled experimental design pre-test/post-test protocol designed to investigate the effects of mental fatigue on neurophysiological, visual, technical, and motor performance in football players. Thirty-four athletes (17 men, 17 women; aged ≥18 years; ≥1-year competitive experience) will be randomly assigned to either a cognitive fatigue induction group (30 min Stroop Task) or a low cognitive load control group. Data collection will include EEG (theta and alpha bands), eye tracking (fixation, saccades, pupil dynamics), sport-specific tasks (Loughborough Soccer Passing Test, Y-Shaped Agility Reaction Test), and subjective fatigue ratings (Fatigue Assessment Scale; Visual Analogue Scale). Statistical analyses will encompass intra- and inter-group comparisons, correlation and regression models. By integrating subjective, physiological, and performance indicators in a sport-specific, ecologically valid design, this protocol aims to deepen understanding of how mental fatigue affects performance and to inform individualized monitoring and intervention strategies in high-performance contexts. As a protocol paper, no data are yet available. However, based on previous literature, mental fatigue is expected to be associated with changes in EEG oscillatory activity, less efficient gaze behaviour, and impaired football-specific performance compared to the control condition.

## 1 Introduction

Mental fatigue is defined as a psychobiological state resulting from prolonged and demanding cognitive activity, leading to a subjective feeling of tiredness, increased perception of effort, reduced motivation, and impaired cognitive and motor performance (1, 2). These changes may lead to declining in the performance of sports tasks requiring cognitive resources, such as rapid decision-making, precise motor execution, and perceptual-motor adaptation, especially under pressurized conditions. Within the cognitive sciences the negative impact of mental fatigue on attention and executive control processes are already established (3). In sporting contexts, similar evidence is accumulating (4, 5). Dynamic sports performance—demanding quick responses, motor precision and rapid decision-making under pressure—may be particularly vulnerable to the effects of mental fatigue. Several studies show that mental fatigue compromises technical performance, reduces reaction speed and impairs motor accuracy in competition (1, 4, 6, 7).

Performance declines, caused by mental fatigue, have been investigated through behavioral, neurophysiological and perceptual measures, with robust evidence, demonstrating impairments in the cognitive and motor sport tasks (1, 3, 7). High cognitively demanding tasks, such as Stroop Task (ST), are frequently applied in experimental protocols for induction of mental fatigue, due to their sustained engagement of attention and inhibition regulation systems. Such tasks also exert detrimental effects on subsequent performance; affecting both technical execution and physical preparedness (4, 8). Moreover, some studies confirm mental fatigue has deleterious effects on reaction time, motor accuracy, and decision-making, in particular when there is a need for rapid adjustments and fast responses under stress (1).

Collectively, existing literature highlights that high energy costs and reduced motivation—driven by prolonged cognitive efforts—are at the root of mental fatigue's negative impact (9–11). These effects include a reduction in reaction time, increasing technical errors, diminishing motor accuracy, and disruption of perceptual-attentional strategies: All important functions for cognitively complex tasks, such as football match-play (2, 12). Recent findings also highlight mental fatigue impairs neuromuscular coordination and cortical excitability during demanding physical efforts, which subsequently influences technical execution and reaction time in sport-specific scenarios (13–15).

From a neurophysiological perspective, mental fatigue often manifests as an increase in theta and alpha power frequency bands, measured by Electroencephalogram (EEG), especially in the frontal region. These changes are associated with reduced vigilance, reduced attentional effort and less efficient executive control (16–18). Some experiments, for example Wascher et al. (19), show that frontal theta activity is highly correlated with specific components of mental fatigue, such as the depletion of attentional resources and sustained cognitive overload. These differences indicate a lower level of vigilance and a decreased information processing efficiency, which may be related to lowered attentional effort and less efficient executive control (20–23). Simultaneously, mental fatigue also compromises visual attention, a critical function underpinning perceptual-motor performance in sports tasks. Eye tracking system (ETS) enable analysis of gaze patterns (e.g., saccades, blink rate, pupil dilation, fixations), which have been validated as objective indicators sensitive to cognitive effort and fatigue status (24, 25). Recent studies show that mental fatigue compromises visual exploration efficiency and delays oculomotor response, with subsequent negative impacts on technical performance and decision-making (26, 27).

In addition, subjective measures have been widely used to capture individual perceptions of mental fatigue, offering a phenomenological perspective that complements physiological and behavioral approaches. Visual Analogue Scales (VAS), for example, are used to quantify momentary variations in fatigue status with high sensitivity and easy application (28, 29). Also, Fatigue Assessment Scale (FAS) demonstrate psychometric robustness in assessing physical and mental dimensions of fatigue and have been validated in different populations and contexts (30–33).

For behavioral assessment of sport performance, specific tools validated in the context of football also stand out. The Loughborough Soccer Passing Test (LSPT) is a standardized task that assesses technical passing accuracy under time pressure, by integrating elements of motor control, decision-making and rapid execution. This assessment has demonstrated validity and reliability in discriminating skill levels in football players and is widely used to monitor technical performance under demanding conditions (34, 35). In turn, Y-Shaped Agility Reaction Tests (Y-SART) enable quantification of agility and reaction speed in response to visual stimuli, and are particularly sensitive to the influence of fatigue on decision-making and rapid changes of direction (36). These performance tests provide objective and ecologically valid measures that could capture the impact of mental fatigue on complex motor tasks in football.

Despite growing interest in the topic, the existing literature exhibits sizeable methodological gaps. Few studies have adopted multimodal approaches that simultaneously integrate electrophysiological (e.g., EEG) and/or oculometry measures, such as those captured by ETS, subjective perceptions of fatigue (e.g., VAS) and motor performance (e.g., LSPT) in a single cohesive experimental structure. Although some studies explore the effects of cognitive load on oculometry parameters [e.g., (25, 37)], few studies simultaneously integrate neurophysiological, visual, subjective and motor measures, hindering a holistic understanding of the impact of mental fatigue in sport context. This fragmentation hinders a comprehensive understanding of the effects of mental fatigue in dynamic sporting contexts that require quick decisions and precise movements.

The absence of integrated protocols limits not only the ecological validity of results, but also their applicability to training and performance contexts. Recently, the importance of more comprehensive and ecologically valid experimental designs—simultaneously integrating assessments of athletes' physical, technical, and cognitive performance –, has been advocated in the literature (7, 27); primarily because mental fatigue diminishes not only physical and technical skills, but also interferes with the attentional resources necessary for efficient execution of football-specific tasks (2, 6, 12).

This study proposes a methodology and an experimental protocol to assess mental fatigue and its effects on physical performance in football. Subjective and objective instruments will be used to assess mental fatigue, namely: FAS, to measure the general level of physical and mental fatigue; VAS, applied at different times to capture moment variations in fatigue perception; ETS, to analyze saccades, blink rate, pupil dilation, and fixation pattern as markers of cognitive overload; and EEG, recording the brains electrical activity, with a focus on frontal theta and alpha bands associated with attention and executive control. Physical performance will also be assessed via two motor performance tests: LSPT, which assesses technical accuracy and decision-making under time pressure; and Y-SART, measuring the agility and reaction time to visual stimuli (38).

There is a clear need for an ecologically-valid, football-specific experimental protocols integrating neurophysiological, visual, subjective and sport-specific motor measures within a single, coherent design. The present protocol describes a randomised controlled pre-post protocol design to examine how acute mental fatigue affects cortical activity, gaze behaviour and technical-motor performance in football players.

This methodology combines EEG, head-mounted eye tracking during the LSPT, validated agility assessment with the Y-SART, and subjective fatigue ratings to provide a multimodal characterisation of mental-fatigue responses. Our hypothesis is that, compared to a low-load control condition, a 30 min ST will induce higher subjective fatigue, altered frontal-parietal EEG activity, less efficient visual exploration, and decrements in football-specific performance. If confirmed, these findings will support the use of this protocol as a replicable framework for research and applied monitoring in high-performance football environments.

## 2 Materials and methods

### 2.1 Study design

**Figure 1:**
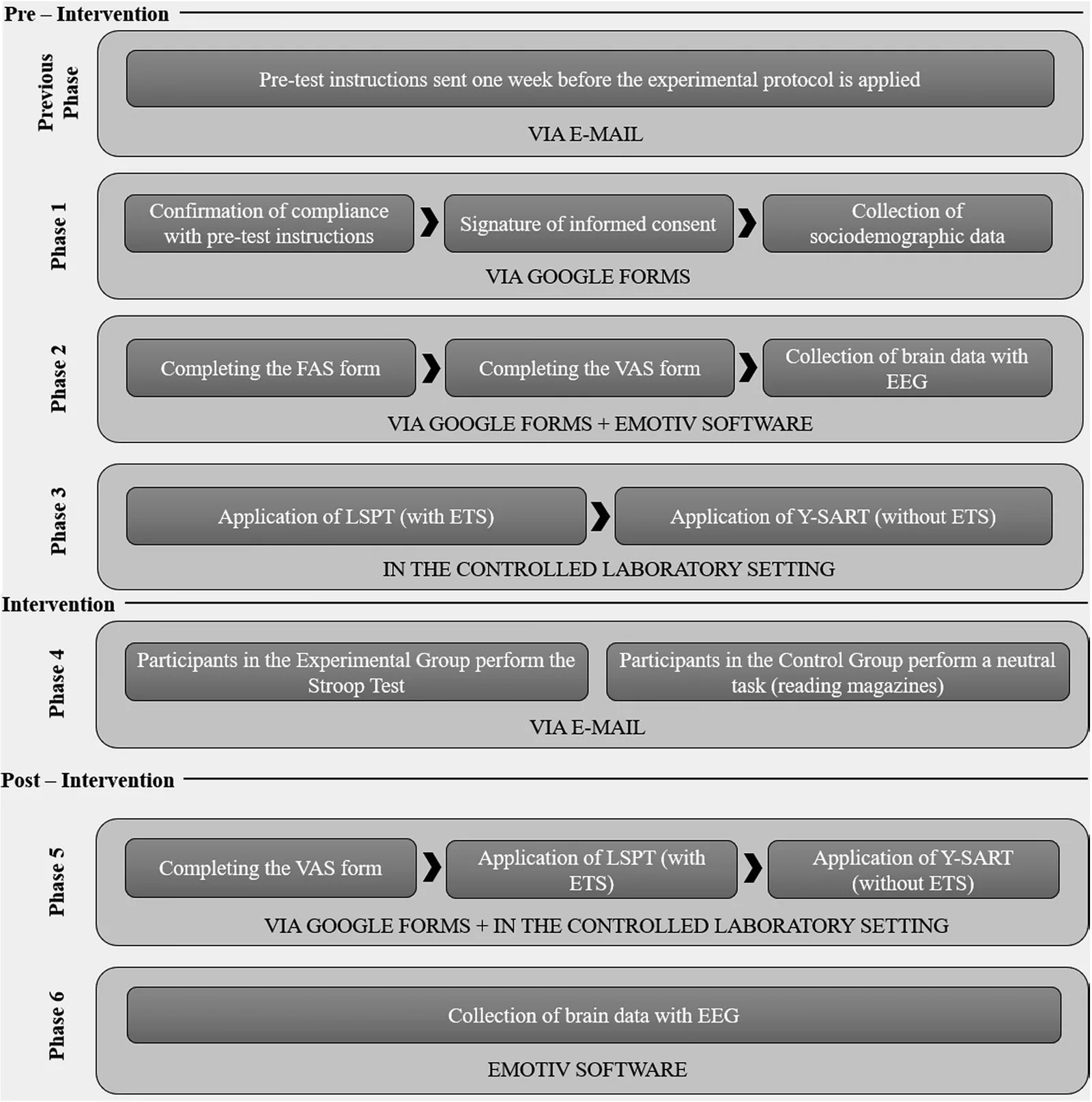
Study design.

This study employed a randomised controlled experimental design, double-group pretest-posttest design, to evaluate the effects of mental fatigue on football players performance (39, 40). Due to its experimental design, the investigation will be carried out in a controlled laboratory setting where cognitive and motor tasks will be standardized and applied. The sequence of procedures and data collection moments is detailed in Figure 1, which outlines the six phases of the methodology and experimental protocol, including both pre and post intervention assessments. Although conducted in a controlled laboratory environment, this design prioritizes internal validity to isolate acute fatigue-related effects while incorporating football-specific performance tasks to preserve ecological relevance.

Ethical approval was granted by the Ethics Committee of Polytechnic University of Coimbra (reference D96/2024), ensuring that the research complies with the ethical standards, with priority given to the well-being and rights of the participants. All procedures are in accordance with the ethical principles established in the Declaration of Helsinki, as well as with the requirements defined by the General Data Protection Regulation of the European Union (GDPR-EU2016/679).

In addition to institutional approval, the study protocol will also be registered in a public clinical trial database, promoting transparency and accountability in the research process. This registration will allow public access to the methodological details of the investigation. By securing both ethical approval and trial registration, the study commits to the principles of ethical conduct in research, ensuring that participant safety and informed consent are respected throughout the process.

### 2.2 Sample

Sample size was calculated using G*Power 3.1.9.7 software (41), considering a repeated measures ANOVA design with two groups (experimental *vs.* control) factors, and two measurement times (pre *vs.* post). The estimated minimum sample size at alpha 0.05 and statistical power of 80%, for moderate effect size (f = 0.25), was approximately 34 subjects (17 per group). At baseline, participants will be pre-screened using an online questionnaire to determine eligibility.

The inclusion criteria were: (i) minimum age of 18; (ii) at least one year of competitive football experience; (iii) no eye problems that impair vision; (iv) no history of neurological injuries; (v) abstinence of caffeine, alcohol and intense physical activities in the previous 24 h before each experimental session; (vi) having slept for at least 7 h the night before. On the other hand, the exclusion criteria were: history of neurological injury; uncorrected vision problems; diagnosis of psychological disorders; use of psychoactive substances in the last 24 h before the experimental sessions; any form of nystagmus for electrophysiological contraindications for electrophysiological recording. The operationalization and description criteria are summarized in Table 1.

**Table 1:** Sample operationalization and description criteria.

Topic	Description	Operationalization
Age	Minimum age of 18 years	Participants aged 18 years or older. There is no maximum age limit stipulated in the protocol.
Gender	Gender balance	Sample of 24 men and 24 women, in equal number for experimental and control groups.
Competitive experience	Minimum of 1 year of competitive experience	Football players with a minimum of 1 year of regular practice in competitive condition
Health condition	No conditions preventing the practice of sport	Individuals with a history of neurological injuries, uncorrected visual problems, diagnosed psychological disorders, use of psychoactive substances in the last 24 h, or any clinical limitation to physical exertion will be excluded. Pregnant women will also be excluded.
Legal and ethical criteria	Compliance with current regulations	The study complies with the GDPR and the provisions of Decree-Law No. 5/2007, ensuring that all participants meet the legal and ethical conditions for physical exercise.

During the previous phase, participants will receive specific instructions by e-mail one week before the experimental session, specifying preparation procedures to ensure standardized test conditions.

Upon arrival at the controlled laboratory setting (Phase 1), participants will first be reminded of the study procedures and objectives. After confirming their understanding, each participant will sign an informed consent form, in accordance with the General Data Protection Regulation (GDPR) and Portuguese legislation (Decree-Law No. 5/2007), ensuring ethical compliance and fitness for sports-related assessments. Adherence to pre-test instructions will then be verified, followed by the administration of a structured questionnaire to collect sociodemographic and sports-related information (age, gender, position on the field, training experience, weekly training load, sleep habits, caffeine intake, and history of injury). This data will be used to contextualize participants and control potential confounding variables. These variables may be considered as exploratory covariates in secondary statistical analyses to examine potential moderating effects of competitive experience and contextual factors on fatigue-related outcomes. However, the study is primarily powered to detect main fatigue-related effects rather than complex interaction models.

Following baseline assessment, all participants will be randomly assigned to either the experimental group or the control group (Phase 4). The experimental group will perform a cognitively demanding task, the ST, for 30 min, designed to induce mental fatigue through sustained attention and inhibitory control demands. Although the ST does not reproduce the full cognitive complexity of real football match situations, it is widely used in experimental research to induce a controlled state of mental fatigue under standardized conditions (4, 8). In the present protocol, it is not intended to simulate football-specific decision-making demands, but rather to induce mental fatigue prior to sport-specific performance assessment. This separation between fatigue induction and performance testing allows clearer interpretation of the neurophysiological and behavioral consequences of mental fatigue. In contrast, the control group will engage in a low-demand relaxation condition, consisting of reading neutral magazines for the same duration (30 min), thereby maintaining minimal cognitive load while controlling for time-on-task effects.

### 2.3 Mental fatigue assessment

The mental fatigue will be assessed using a multidimensional approach, employing different complementary methods. These will include validated subjective instruments such as the FAS and the VAS, which will be administered digitally during Phase 2. The FAS, developed by Michielsen et al. (42) and validated for the Portuguese population by Alves and Nazaré (43), is recognized for its reliability, presenting high internal consistency (α = 0.870). It consists of 10 items on a 5-point Likert scale (1 = never, 5 = always) and assesses the perception of general fatigue. The items question the frequency of physical and mental symptoms of fatigue. This scale will only be applied at the beginning of the session, as it provides a stable measure of the general state of fatigue, suitable for the participants initial characterization. In turn, the VAS will be applied before and after the cognitive task, given its sensitivity to momentary variations in perceived mental fatigue. It is a single-item measure that asks participants to indicate the intensity of fatigue felt at the moment, by marking a point on a continuous line delimited by two extremes (no fatigue—extreme fatigue). For analysis, the marked position is converted into a continuous numerical measure (0–100). The use of this scale in Portugal is supported by national literature, namely in the studies by Afonso et al. (44) and Alves & Nazaré (43). Both will be administered digitally through the Google Forms platform, ensuring standardization and efficiency in data collection.

In parallel, during Phase 2, brain electrical activity will be recorded using the portable Emotiv Epoc Flex Gel Kit system, equipped with Ag/AgCl sensors arranged according to the international 10–20 system (32 channels), with reference and ground electrodes placed in standard positions. Data will be sampled at 128 Hz, ensuring impedances below 10 kΩ to guarantee signal quality. Each recording will have a total duration of five minutes, divided into three conditions: eyes open (two minutes), eyes closed (two minutes), and motor imagery (one minute). Participants will sit comfortably in a quiet environment to avoid external interference or movements that could generate artefacts. The selection of these three conditions is supported by evidence demonstrating that distinct neural states can be captured through resting conditions and motor imagery paradigms (45–47). Eyes-open and eyes-closed recordings are known to reflect different oscillatory dynamics, particularly in the alpha and theta bands (48, 49), while motor imagery reliably evokes measurable cortical activation and is sensitive to mental fatigue (50–52). In the statistical analyses, recording condition (eyes open, eyes closed, motor imagery) will be treated as a within-subject factor, allowing evaluation of whether fatigue-related changes differ across neural states.

To ensure data quality and reproducibility, EEG signals will undergo standardized pre-processing procedures. Data will be processed in MATLAB R2025a (MathWorks, Natick, MA, USA) using EEGLAB v2025. Continuous recordings will be visually inspected to identify gross artifacts or recording instabilities, and non-EEG channels will be removed prior to analysis. Signals will be band-pass filtered between 1 and 45 Hz using a zero-phase finite impulse response (FIR) filter and re-referenced to a common average reference. Artifact correction will be performed using Independent Component Analysis (extended infomax algorithm). Independent components will be automatically classified using the ICLabel plugin integrated in EEGLAB, and components identified as ocular, muscular, cardiac, line-noise, or other non-brain sources with high probability (≥0.90) will be removed prior to signal reconstruction. Channels exhibiting persistent excessive noise throughout the recording will be excluded from analysis. All preprocessing procedures will be applied consistently across participants and experimental conditions to ensure methodological standardization and internal validity.

Oculomotor activity will be recorded with the Pupil Core system (Pupil Labs GmbH, Germany), with sampling at 200 Hz. This equipment will allow the analysis of fixations, saccades, pupillary dilation or and blink rate, during the LSPT. The ETS was only applied in the LSPT, as it contains visual stimuli while performing gaze orientation and control of attention, appearing as especially appropriate to analyze gaze patterns (53). In addition, the LSPT is longer in duration, which makes it possible to collect consistent eye activity data. Vansteenkiste et al. (54) demonstrated a correlation between LSPT performance and gaze patterns in young players, reinforcing that this type of test is suitable for analyzing oculomotor metrics. In contrast, the Y-SART is a simple task and it presents brief visual stimuli, so the use of an eye-tracker is not justified. Recent research has demonstrated that motor skills (e.g., sprinting, changing direction, reactive strength) are the main determinant in performance on the Y-SART and is also affected by sensory/perceptual processes to a smaller extent or indirectly (55). The data will be processed with open-source software provided by the manufacturer.

### 2.4 Performance assessment

**Figure 2:**
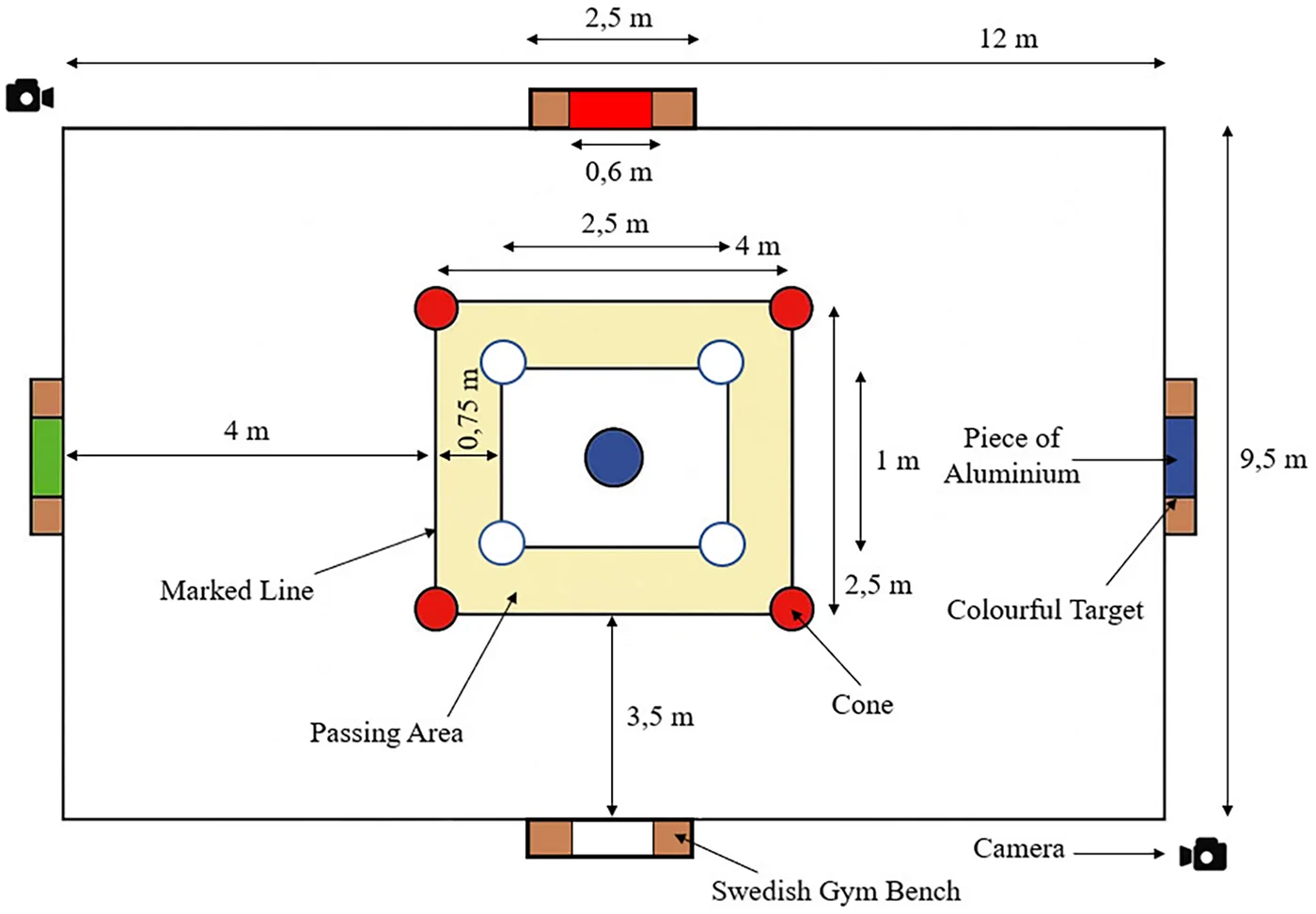
Schematic representation of the LSPT, adapted from Ali et al. (34).

After collecting baseline data, participants will perform two motor tests as part of Phase 3 of the protocol. The first is the LSPT, which assesses passing accuracy and speed under time pressure in a standardized soccer-specific context. Participants perform 16 passes to designated targets announced in sequence, with performance assessed by total execution time adjusted for penalty points for technical errors (e.g., incorrect passes, errors in the target area, poor ball handling) and bonus points for accurate actions. This scoring protocol has been validated in youth and elite football players (56, 57). The test will be filmed with two cameras for subsequent technical analysis and penalty verification. A schematic representation of the LSPT setup is provided in Figure 2, adapted from Ali et al. (34).

**Figure 3:**
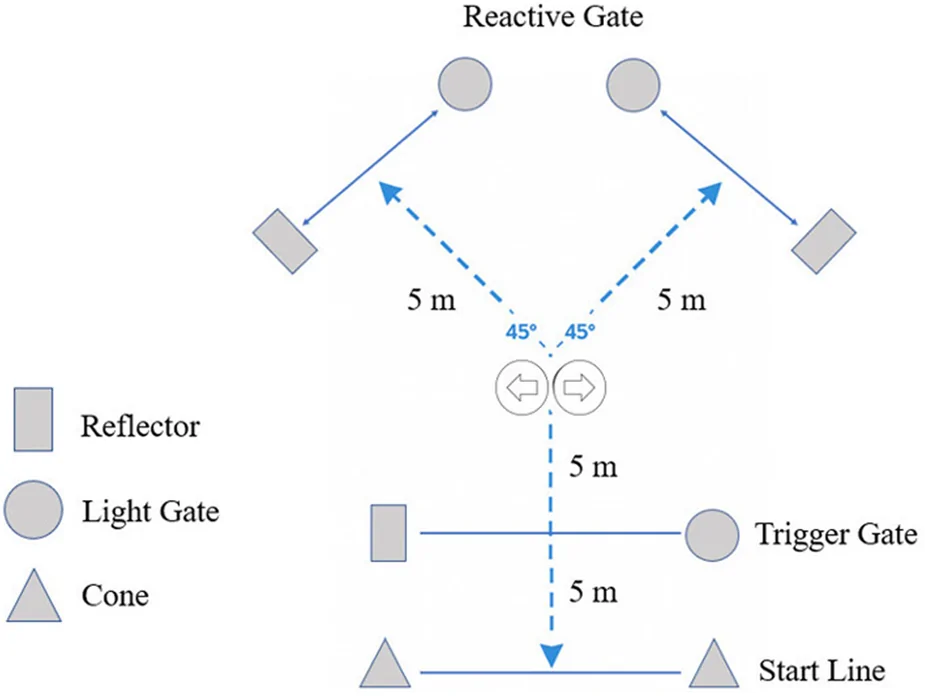
Schematic representation of the Y-SART, adapted from Yuan et al. (2), licensed under CC BY-NC-SA 4.0.

The second physical performance test is the Y-SART, designed to assess agility and reaction time to visual stimuli. Participants start the course from a fixed point and, after an initial start, must react to a visual stimulus provided by the WITTY SEM system, which indicates the need to deviate to the left or right on the Y-SART course. Times are automatically recorded from the start to the finish line, ensuring accurate measurement of reaction and movement speed. A schematic representation of the Y-SART configuration is also provided, in Figure 3, adapted from Yuan et al. (2).

After completing Phase 3, participants move on to Phase 4, where they perform the cognitive intervention defined by their group assignment, either the ST (experimental group) or a neutral task, such as reading magazines (control group). This is followed by Phase 5, which begins with participants completing the VAS again. Next, both motor tests are repeated, this time with continuous recording by the ETS. Finally, a second EEG recording is performed under the same conditions as in the initial phase, concluding the data collection session.

### 2.5 Outcomes

The outcomes defined in this protocol aim to quantify, in an integrated manner, the effects of mental fatigue on different domains of football performance, covering neurophysiological, oculometric, motor, and subjective variables. The definition of these outcomes allows for a multimodal analysis, based on objective and subjective indicators, which reflect the complexity of cognitive and motor responses under conditions of mental overload. This section describes the methodological framework and outcome measures designed to assess the multidimensional effects of mental fatigue in the neurophysiological, oculomotor, motor, and subjective domains.

#### 2.5.1 Cortical activity (EEG)

Cortical electrical activity will be measured with the EMOTIV EPOC Flex EEG device, in order to assess neurophysiological changes related to mental fatigue elicited by the ST. Validation studies have demonstrated that the Emotiv system is capable of recording physiologically meaningful EEG signals and event-related potentials comparable to laboratory systems, although with lower signal-to-noise ratio and reduced reliability compared to medical-grade devices (58, 59). These findings suggest that while suitable for non-critical research applications, limitations remain when high precision or maximal signal robustness is required.

The analysis will focus on the theta (4–7 Hz), alpha (8–13 Hz) and beta (13–30 Hz) frequency bands, with an emphasis on the frontal and parietal regions, traditionally linked through processes of executive control, sustained attention, sensory-motor integration and regulation of the mental load (19, 60–62).

Although increases in frontal theta power and decreases in parietal alpha power have frequently been reported in association with sustained cognitive effort and fatigue-related states, these oscillatory changes are not specific or exclusive markers of mental fatigue. Theta and alpha modulations are influenced by multiple cognitive and attentional processes, including task engagement, vigilance, motivational state, and general mental workload (19, 60, 61). In addition, alterations in beta activity have been associated with changes in cortical stability and motor-related activation (61, 62). Therefore, in the present protocol, changes in these frequency bands will be interpreted as neurophysiological correlates associated with cognitive load and fatigue-related states, rather than definitive biomarkers of mental fatigue.

##### 2.5.1.1 Selection and justification of EEG channels

Electrodes F3, F4, Fz, C3, C4, Cz, P3, P4, Pz, T3, T4, T5, T6, O1, O2, and Oz will be analysed according to the international 10–20 system, allowing comprehensive coverage of the frontal, central, parietal, temporal, and occipital regions. The frontal, central, and parietal regions are particularly sensitive to the effects of mental fatigue, showing increases in theta band power and reductions in alpha band power, phenomena associated with greater attentional effort and decreased cognitive efficiency (19, 61, 62). The temporal and occipital regions were included to ensure a comprehensive characterisation of cortical activity, as previous studies have identified changes in functional connectivity and neural complexity associated with sensory integration and visuospatial regulation under fatigue (62).

Based on explicit theoretical hypotheses, frontal midline electrodes (particularly Fz and adjacent frontal sites) are defined as primary regions of interest (ROIs), given the well-established association between frontal theta activity and mental fatigue. Posterior parietal-occipital regions are included due to their documented involvement in alpha modulation and attentional processing under cognitive load. Analyses focusing on these predefined ROIs and frequency bands (theta, alpha, beta) will be considered primary. Analyses involving additional scalp regions will be clearly identified as exploratory and interpreted with appropriate caution.

##### 2.5.1.2 Spectral analysis

EEG recordings will be acquired before (Pre) and after (Post) the performance of motor tasks, allowing the evaluation of the modulation of the global cortical state between moments and experimental conditions (experimental group vs. control group) (62). The EEG recordings will be obtained under resting-state conditions before and after mental fatigue induction. As no stimulus-locked tasks are included during EEG acquisition, event-related potential (ERP) analyses are not applicable within the present design. Although full-spectrum EEG data (delta, theta, alpha, beta, and low gamma) will be acquired across all electrodes, primary analyses will focus on theta, alpha, and beta power, given their consistent association with mental fatigue in previous literature (19, 61). Connectivity-based analyses are considered beyond the scope of this protocol and may be explored in future investigations. Between group comparisons will be conducted to detect whether the ST elicits greater alterations in alpha and theta power than the control condition, thus establishing whether different cognitive demands produce distinct neurophysiological signatures of fatigue. Comparisons between moments and groups will allow the identification of cortical alteration patterns associated with fatigue-related cognitive states. However, these oscillatory modulations will be interpreted in conjunction with behavioral, oculomotor, and subjective measures, acknowledging that EEG frequency changes reflect multifactorial neural dynamics rather than isolated biomarkers of mental fatigue (19, 60, 63, 64).

Within the experimental group, pre- and post-task EEG analyses will assess the impact of the ST on cortical activation, particularly changes in frontal theta and parietal alpha bands, to capture acute neurodynamic changes resulting from cognitive overload. Recent evidence by Krukow et al. (65) supports this approach, having demonstrated that mental fatigue states are reliably associated with altered EEG connectivity and spectral profiles, especially in the theta and alpha bands, during cognitively demanding tasks. These within-group comparisons aim to detect functional adaptations linked to increased attentional effort, executive control demands, and potential declines in motor preparation efficiency.

In addition to these comparisons, correlational analyses will explore the relationships between EEG parameters and behavioral performance. Power values in the relevant bands will be examined in relation to LSPT and Y-SART results, with the aim of identifying neurophysiological predictors of motor performance under conditions of fatigue.

Furthermore, multimodal associations will be conducted to determine the degree of alignment between EEG data and other indicators of fatigue, namely the VAS and oculomotor metrics captured through the ETS. This triangulation seeks to assess the convergence between subjective, neurophysiological, and visual-attentional markers of mental fatigue.

Topographic maps (topo plots) of cortical distribution by band and experimental moment will also be generated, allowing the spatial location of power changes in the scalp to be visualized and the neurophysiological dynamics associated with mental fatigue to be characterized (15, 19, 60, 66, 67). These integrated approaches can support the identification of electrophysiological biomarkers of fatigue that reflect the brain's adaptation to cognitive effort and its consequences for motor readiness in sports contexts.

In addition, access to large EEG databases, such as the Motor Movements/Images Dataset recently organized by Shuqfa et al. (68), has driven the development of methods for classifying and analyzing real and imagined motor tasks, enabling more accurate calibrations and informing decisions about the cortical bands and locations to be monitored in experimental protocols applied to sports contexts.

#### 2.5.2 Oculomotor parameters (ETS)

Oculomotor parameters will be monitored using the Pupil Pro Eye Tracking Headset (Pupil Labs GmbH, Germany) to assess neurophysiological changes associated with mental fatigue induced by the ST. The metrics analysed will include the number and average duration of fixations, the duration and average speed of saccades, pupil diameter, and blink rate, parameters widely recognized as sensitive indicators of cognitive load, attention regulation, and fatigue-related decline in vigilance (69–72). To minimize potential confounding influences, standardized task instructions, calibration procedures, controlled lighting conditions, and consistent testing environments will be implemented. Moreover, the within-subject pre-post design allows each participant to serve as their own control, thereby reducing the impact of inter-individual variability in visual strategies and perceptual tendencies.

In addition to descriptive characterisation, the study will include comparisons between the experimental and control groups in terms of these oculomotor parameters after the intervention phase. These comparisons will assess whether mental fatigue induced by the ST produces significant changes in visual-attentional dynamics compared to a cognitively neutral task. Specific attention will be given to variations in blink rate, pupil diameter, fixation duration, and saccade characteristics.

Within the experimental group, oculomotor parameters will be compared before and after the intervention to identify the direct impact of cognitive overload on visual attention and eye movement control. This intragroup analysis is crucial for understanding the dynamic response of the oculomotor system to cognitive stress.

In addition, correlational analyses will be performed between ETS data and behavioral performance on the LSPT and Y-SART to explore whether visual-attentional indicators predict motor performance under fatigue. A multimodal approach will also be employed to assess convergence between different markers of fatigue (ETS, EEG, and VAS), providing a comprehensive understanding of how different modalities reflect fatigue-related changes.

##### 2.5.2.1 Fixations

The average duration of fixations reflects the time required for visual information processing and is directly associated with the allocation of attentional resources. Under mental fatigue, the dynamics of fixations become less efficient, typically characterized by longer fixation durations and a reduced number of fixations, reflecting slower cognitive processing and reduced efficiency in visual exploration. Empirical evidence confirms that the proportion of longer fixations (>150 ms) increases significantly under fatigue, while short fixations (<150 ms) decrease, indicating a compensatory adjustment of attention to maintain task performance despite cognitive decline (72, 73).

##### 2.5.2.2 Saccades

Saccades, which represent rapid movements between fixation points, are another relevant indicator of the functional state of the oculomotor system. Given that saccadic eye movements are closely related to neural activity in the frontal lobe, it is highly likely that the use of saccades as a biomarker of mental fatigue will demonstrate high reliability and validity (63). Mental fatigue is often associated with a reduction in the speed and duration of saccades, as well as an increase in variability between successive movements, reflecting a decrease in the neural excitability of visuomotor structures and less efficient attentional control (72, 74).

##### 2.5.2.3 Pupil diameter

The average pupil diameter is a reliable marker of the level of autonomic activation and cognitive load. During the performance of demanding tasks, initial pupil dilation is observed, associated with attentional effort and noradrenergic activation, followed by progressive contraction as mental fatigue develops, reflecting a decrease in locus coeruleus activity and the ability to mobilize cognitive resources (75, 76). Under controlled lighting conditions, the pupil diameter is generally between 3.5 and 4.5 mm when alert, and can reach about 5 mm during periods of high cognitive load. As mental fatigue sets in, a progressive reduction to values close to or below 3 mm is observed, reflecting lower phase variability and pupil responsiveness, as well as a reduction in locus coeruleus activity and cortical vigilance (72, 75, 76).

##### 2.5.2.4 Blink rate

The blink rate is a psychophysiological marker sensitive to variations in cognitive load, mental fatigue, and dopaminergic activity. During periods of high cognitive demand, the blink rate tends to decrease significantly, reflecting intensified attentional control and voluntary inhibition of the blink reflex in order to avoid visual interruptions during information processing. However, as mental fatigue sets in, a progressive increase in the blink rate is observed, indicative of a compensatory collapse of the oculomotor system and a reduction in cortical vigilance (72, 77–79). On average, the blink rate is between 15 and 25 blinks per minute (≈0.25–0.42 Hz) in a neutral state, decreasing to 5–15 blinks/min (≈0.08–0.25 Hz) during cognitively demanding tasks, and increasing again to values above 25–30 blinks/min (≈0.42–0.50 Hz) in advanced stages of mental fatigue. This biphasic pattern is widely described as an expression of dopaminergic modulation underlying initial attentional effort and subsequent reduction in cortical vigilance (72, 78).

#### 2.5.3 Technical and motor performance

Technical and motor performance will be assessed using the LSPT and the Y-SART, using Witty SEM indicators (Microgate, Bolzano, Italy). These instruments allow the effects of mental fatigue on technical execution, reactive agility and decision-making processes in a sporting context to be quantified with high temporal precision and reliability.

Comparisons between groups will examine whether the ST induces greater impairments in performance metrics compared to a cognitively neutral task. The LSPT will provide data on total execution time, technical penalties, and adjusted score, while the Y-SART will produce reaction and execution times in response to visual stimuli. These comparisons will elucidate the degree to which mental fatigue affects motor fluency, visuomotor coordination, and accuracy under pressure.

Intra-subject analyses in the experimental group will allow for the detection of performance deterioration caused by the ST. By comparing pre- and post-intervention scores on both the LSPT and Y-SART, this approach will help determine whether mental fatigue results in statistically significant reductions in motor and technical efficiency.

The associations between performance metrics and indicators of fatigue (e.g., VAS, EEG, and ETS) will also be examined to explore whether higher levels of perceived or physiological fatigue correlate with slower and less accurate performance. These relationships may help identify which dimensions of fatigue are most predictive of declines in physical performance.

##### 2.5.3.1 LSPT

In the LSPT, the variables total execution time, technical penalties, and adjusted score will be analysed, indicators that simultaneously integrate motor speed, technical accuracy, and cognitive efficiency under time pressure. This protocol demonstrates high ecological validity and excellent test-retest reliability for assessing technical proficiency and decision-making performance in football (4, 57). Recent evidence shows that mental fatigue leads to an increase in total time and penalty time, indicating that mentally fatigued athletes are more prone to technical errors, such as missing target zones. This decline in performance appears to stem from a shift in attention towards irrelevant stimuli rather than task-relevant stimuli, resulting in poorer technical execution and slower motor fluency (80, 81).

##### 2.5.3.2 Y-SART

The Y-SART will use the Witty SEM system to deliver random light stimuli requiring rapid directional responses. Reaction and execution times will be recorded as indicators of visuomotor coordination and decision-making efficiency under cognitive load. Under conditions of mental fatigue, an increase in reaction time is expected in the Y-SART, reflecting slower perceptual-decision processing and reduced cognitive readiness, while execution time may also show moderate increases due to delayed motor initiation. This pattern is consistent with the evidence that reactive agility performance is particularly sensitive to cognitive overload and fatigue effects (82).

In line with Khalaji et al. (14), who reported impairments in decision-making accuracy and execution speed following mental fatigue induction, the present study employs the LSPT and Y-SART to assess the behavioral impact of cognitive overload in football contexts.

Although the protocol is conducted in a controlled laboratory setting, the selected football-specific tests require multidirectional actions and quick responses to external cues, partially reflecting perceptual and decision-making demands present in match play. The controlled environment helps reduce external influences while maintaining key sport-specific elements.

#### 2.5.4 Subjective fatigue measures

Individual perceptions of fatigue will be assessed using the FAS and the VAS, both validated instruments for quantifying subjective mental and physical fatigue.

##### 2.5.4.1 FAS

The FAS provides an overall measure of perceived fatigue, covering both physical and mental components. This 10-item unidimensional scale has demonstrated strong internal consistency (α = 0.71–0.89) and excellent test-retest reliability in populations including clinical and athletic samples (32, 42, 83). Its Likert format (1 = “never” to 5 = “always”) allows for the assessment of cognitive, motivational, and energy-related aspects of fatigue, providing a reliable index of baseline fatigue levels. This measure will be used in both the experimental and control groups, allowing comparisons of baseline mental fatigue prior to the Intervention.

##### 2.5.4.2 VAS

The VAS for mental fatigue, applied immediately before and after the cognitive induction task (ST), captures acute fluctuations in the subjective perception of fatigue. This 100 mm continuous scale, anchored between “no fatigue” and “extreme fatigue”, is sensitive to short-term changes induced by cognitive load and task duration (84). Within the experimental group, VAS ratings will be compared pre and post ST to quantify the immediate impact of the intervention on mental fatigue.

In addition, associations between subjective fatigue and other objective markers will be explored. Specifically, post-intervention VAS results will be correlated with EEG spectral characteristics (particularly alpha and theta power), oculomotor parameters (blink rate, pupil diameter, saccadic dynamics, and fixations), and motor performance measures (LSPT and Y-SART). This approach allows us to determine whether higher levels of self-reported fatigue align with neurophysiological and behavioral indicators of reduced performance, providing a multimodal framework for validating the perceptual dimension of fatigue.

#### 2.5.5 Individual variability

Interindividual variability in responses to mental fatigue will be analysed to identify personal and sporting factors that modulate the impact of cognitive overload on neural, visual, and motor performance. This analysis will help clarify how specific athlete profiles cope with mental fatigue and maintain performance efficiency under cognitive stress.

Demographic and contextual variables such as age, gender, field position, training experience, sleep habits, caffeine consumption, and injury history will be considered. These parameters will be used as exploratory covariates to examine their moderating effect on the changes observed in the EEG, ETS, LSPT, and Y-SART results. Recent literature indicates that such individual factors modulate susceptibility to fatigue and help explain the variability in cognitive and physical performance under mental load (10, 13, 85).

This analysis will enable the differentiation of profiles that are more or less sensitive to mental fatigue, helping to map individual patterns of cognitive and physiological response to prolonged cognitive activity. It is expected that athletes with higher levels of competitive experience and attention self-regulation will show smaller decreases in performance and smaller neurophysiological changes. This personalized approach, integrated with objective and subjective data, will contribute to the development of individualized strategies to optimize cognitive and neuromotor resilience in demanding sporting environments.

While individual characteristics will be considered as exploratory covariates, the present sample size and experimental structure are primarily oriented toward detecting overall fatigue-related effects rather than complex interaction models between multiple individual variables. Future studies specifically designed to recruit more homogeneous samples (e.g., athletes from the same competitive level or age group) may allow a more in-depth analysis of interindividual variability in mental fatigue responses.

#### 2.5.6 Multimodal integration

The data collected through EEG, ETS, motor tests (LSPT and Y-SART) and subjective measures (FAS and VAS) will be integrated into a multimodal and correlational analysis, with the aim of identifying convergent patterns of response to mental fatigue. This approach allows for the simultaneous examination of cortical, oculomotor, behavioral, and perceptual changes, providing a more complete characterisation of the neurophysiological and functional mechanisms underlying mental fatigue in athletes. Recent studies demonstrate that the integration of multiple measurement modalities, such as EEG, ETS, and performance parameters, improves sensitivity in detecting states of fatigue and understanding its cognitive and motor manifestations (62, 86, 87).

Multimodal analysis will be performed both at the group level (experimental vs. control) and at the intra-subject level (pre- vs. post-intervention). Comparisons between groups will help determine the discriminatory power of each modality in detecting fatigue, while intra-individual assessments will examine how each marker evolves in response to cognitive load. In addition to identifying response patterns across modalities, the study will examine associations between modalities, such as correlations between EEG (e.g., alpha/theta power), ETS markers (e.g., blink rate, saccades), motor performance metrics (LSPT and Y-SART), and subjective measures (VAS, FAS). This integrated framework allows us to assess whether neurophysiological changes are aligned with behavioral and perceptual data, increasing the validity of fatigue assessment.

This multimodal integration also enables the identification of potential short-term compensatory responses. For instance, preserved motor performance accompanied by increased perceived fatigue or altered cortical and oculomotor activity may reflect greater cognitive effort to maintain task execution under mental fatigue. Such patterns may indicate adaptive adjustments in response to cognitive overload, even in the absence of overt performance decline.

This triangulation approach is expected to reveal composite markers of mental fatigue, allowing us to combine EEG data, eye-tracking parameters, motor performance outcomes, and self-reported measures into multidimensional fatigue profiles. Such profiles may inform the development of more accurate monitoring protocols in high-performance environments.

Ultimately, the combined interpretation of these results will provide an integrative understanding of the neurocognitive and behavioral mechanisms underlying mental fatigue in football. This holistic view may contribute to practical strategies for detecting, monitoring, and mitigating fatigue in sporting contexts that require sustained attention and decision-making under pressure.

### 2.6 Statistical analysis

All statistical analyses will be performed using IBM SPSS Statistics version 29.0 (IBM Corporation, Armonk, NY, USA), with a significance level set at 5% (*p* < 0.05). Descriptive statistics (mean, standard deviation, 95% confidence intervals, and range) will be calculated for all outcome variables, including EEG spectral powers, oculomotor parameters, motor performance scores, and subjective fatigue ratings. These data will be stratified by group (experimental and control) and time (pre- and post-intervention) and presented visually using box plots and line graphs to facilitate interpretation (88, 89).

To assess the effect of mental fatigue, paired t-tests will compare pre- and post-intervention results within each group, while independent t-tests will compare the experimental and control groups at each time point (88, 90). For EEG outcomes, repeated-measures ANOVA models will include Time (pre vs. post mental fatigue) and Recording Condition (eyes open, eyes closed, motor imagery) as within-subject factors, and Group (experimental vs. control) as a between-subject factor (88, 90). Assumptions of normality and sphericity will be evaluated prior to analysis, following established recommendations for repeated measures designs (88, 91). When sphericity is violated, the Greenhouse-Geisser correction will be applied. Effect sizes will be reported using eta squared (*η*^2^), interpreted according to Marôco (90) as small (<0.04), moderate (0.04–0.25), or large (≥0.25). Statistical power (π) will also be reported when applicable (92, 93).

For EEG data (theta, alpha, and beta bands), spectral power within predefined regions of interest (ROIs) will be analyzed using repeated-measures models including Time (pre vs. post mental fatigue) and Recording Condition (eyes open, eyes closed, motor imagery), allowing evaluation of fatigue-related changes across neural states. Primary analyses will focus on frontal midline and posterior ROIs, consistent with the study's theoretical hypotheses. Exploratory analyses across additional scalp regions will be conducted separately and corrected using False Discovery Rate (FDR) procedures to control for multiple comparisons. Correlational analyses (Pearson's r) will explore associations between EEG bands and behavioral performance (LSPT, Y-SART), as well as subjective fatigue scores (VAS and FAS) (94, 95). Topographic maps will be generated to visualize EEG power distributions across cortical regions.

For oculomotor parameters (fixations, saccades, pupil diameter, blink rate), comparisons will assess the impact of mental fatigue on attention dynamics and visual search efficiency. Pearson correlations will explore links between ETS metrics, EEG patterns, and motor performance scores. Eye-tracking outcomes will be interpreted in conjunction with EEG measures, motor performance variables, and subjective fatigue scores, allowing a multimodal framework that reduces interpretative ambiguity.

Motor performance will be analyzed by comparing LSPT and Y-SART results before and after cognitive induction. Metrics such as execution time, technical penalties, and reaction times will be examined to determine whether cognitive fatigue impairs decision-making and technical execution under pressure.

These performance results will be correlated with neurophysiological and oculomotor indicators to investigate predictive relationships. Subjective fatigue will be analyzed using pre and post VAS comparisons and baseline FAS scores. These will be used to triangulate subjective perceptions with objective changes in EEG, ETS, and performance data. Higher self-reported fatigue scores are expected to align with reduced cortical efficiency, altered visual behavior, and degraded motor output.

To address individual variability, demographic and contextual variables (e.g., age, gender, field position, experience, sleep habits, caffeine intake) will be tested as covariates using ANCOVA or subgroup analyses. This will help determine whether certain profiles exhibit greater resilience or vulnerability to mental fatigue (10, 96).

Finally, multimodal integration will involve constructing correlation matrices and regression models to identify fatigue profiles across domains. This will allow for the identification of converging patterns across the EEG, ETS, motor, and subjective domains, thereby increasing the understanding of the neurocognitive and behavioral effects of mental fatigue. This approach is supported by advanced multivariate methodologies in sports neuroscience and applied psychology (39, 40).

## 3 Discussion

Although the multimodal approach proposed in this protocol is relatively unexplored in sports science, emerging literature increasingly recognizes the detrimental effects of mental fatigue on attention regulation, decision-making processes, and motor performance, especially in tasks that require cognitive control and rapid perceptual-motor adjustments. Mental fatigue has been associated with increased perceived exertion, decreased motivation, and poorer cognitive control, ultimately leading to a decline in performance in both endurance sports and intermittent disciplines such as football (1, 7). These impairments are believed to stem more from central mechanisms than from peripheral physiological deterioration, such as altered cortical excitability and attention depletion (3, 9). If our hypothesis is confirmed, then mental fatigue will compromise motor and decision performance, as previously shown in sport-specific cognitive-motor protocols (2, 14).

The use of subjective measures, such as the FAS and the VAS, increases the ecological validity of the protocol. The FAS offers a multidimensional assessment of fatigue, covering physical and cognitive domains, and has demonstrated robust psychometric properties in clinical and non-clinical populations (32, 42). Its applicability in sport has been reinforced in recent validation efforts in Portuguese samples (42, 43). On the other hand, VAS is widely recognized for its sensitivity to immediate changes in perceived exertion and cognitive fatigue during highly demanding tasks (28, 29). Evidence also suggests that these subjective tools reliably reflect internal cognitive states, even in the absence of obvious physiological changes, thus offering complementary insights to neurophysiological and behavioral indicators (31, 33). By triangulating FAS, VAS, EEG, ETS, and performance outcome data, the protocol provides a multidimensional lens for examining the experiential and functional impacts of mental fatigue in football context.

The integration of EEG into this protocol is supported by recent evidence demonstrating that mental fatigue causes measurable changes in brain activity, particularly in oscillatory patterns linked to attention regulation and cognitive control. Notably, increased frontal theta power and decreased parietal alpha power have been repeatedly associated with sustained mental effort and attention decline (21, 48, 52). These neurophysiological signatures are especially evident during tasks that require prolonged executive engagement, such as those that mimic competitive sports conditions. Boord et al. (45) and Ke et al. (47) highlighted that mental fatigue leads to a reduction in task-specific cortical differentiation, impairing the brain's ability to maintain optimal states of attention. Di Rienzo et al. (50) and Gwon et al. (51) further demonstrated how EEG spectral analysis can sensitively capture the progressive onset of fatigue over time, even under motor imagery and dual-task conditions. Lum et al. (49) and Yang and Ren (23) suggested that EEG markers may vary depending on individual cognitive resilience and workload history, while Wascher et al. (19) provided initial experimental evidence linking increases in frontal theta to sustained attention overload. Furthermore, Rakhmatulin (17) reported EEG-based reductions in attention efficiency in mentally fatigued athletes, confirming the utility of EEG in sports contexts. These findings justify the inclusion of EEG in this protocol as a central tool for examining the cortical dynamics underlying mental fatigue and its relationship to impairments in visual and behavioral performance in football.

Importantly, although these oscillatory patterns are frequently observed under mental fatigue, they are not specific to fatigue alone and may reflect broader mechanisms related to attention regulation, motivational state, and cognitive workload. For this reason, the present protocol adopts a multimodal interpretative framework integrating EEG findings with behavioral, oculomotor, and subjective measures. Previous work has emphasized that alpha and theta oscillations are modulated by multiple cognitive and attentional processes and should not be interpreted as specific biomarkers of fatigue [e.g., (97, 98)]. Accordingly, in the present study, spectral changes are interpreted as context-dependent neurophysiological correlates rather than direct indicators of mental fatigue *per se*.

In this context, preserved behavioral performance accompanied by increased perceived effort or altered cortical oscillatory activity may be interpreted as short-term compensatory mechanisms aimed at sustaining task execution under mental fatigue. Such patterns can indicate increased cognitive resource allocation despite stable overt performance. Although the present protocol does not specifically model long-term compensatory adaptations, these mechanisms are acknowledged as relevant for future longitudinal investigation.

The inclusion of ETS is supported by growing evidence linking visual behavior to cognitive load and performance efficiency in sporting contexts. Mental fatigue has been shown to disrupt oculomotor dynamics, leading to longer fixation durations, fewer saccades, and altered pupillary responses, which are patterns associated with decreased attentional flexibility and compensatory cognitive effort (25, 26). In sport-specific contexts, ETS provides valuable insights into how athletes distribute visual attention during decision-making under pressure. For example, Zeuwts et al. (27) and Vansteenkiste et al. (54) demonstrated that more effective gaze strategies, characterized by shorter fixations and increased anticipatory saccadic movements, are associated with superior performance in passing and decision-making tasks in football. Crowe et al. (24) further highlighted how increased cognitive demands alter gaze fixation behavior, suggesting that visual metrics may sensitively reflect cognitive fatigue. Furthermore, Souto and Kerzel (53) emphasize the interdependence between selective visual attention and oculomotor control, underscoring the relevance of ETS measures in studies examining attentional resource allocation under fatigue. Collectively, these findings reinforce the utility of ETS for detecting subtle changes in visual search patterns that emerge from mental fatigue and affect performance.

The integration of sport-specific performance tasks into this protocol, namely the LSPT and the Y-SART, is based on their proven ability to assess technical execution and cognitive-motor integration under conditions of decision-making and physical demand. The LSPT has been widely validated to measure passing accuracy, decision-making, and technical skill in football, with proven reliability across different playing surfaces and age groups (34, 35). Le Moal et al. (56) further reinforces its ecological validity, highlighting its sensitivity to training status and competitive level in young players. Similarly, Y-SART provides a robust framework for assessing reactive agility and perceptual-motor responsiveness, essential attributes in football performance. Yuan et al. (2) demonstrated its effectiveness in capturing fatigue-induced declines in decision speed and movement accuracy, underscoring its value in mental fatigue paradigms. Trecroci et al. (36) also emphasized the test's discriminatory power between elite and sub-elite young players, supporting its relevance for talent identification and development profiling. Wang et al. (57) showed that reactive agility tests such as the Y-SART are particularly sensitive to cognitive load, with performance impairments linked to delays in stimulus processing and motor execution. Collectively, these two tests allow for the assessment of sport-relevant behaviors under ecologically valid and experimentally controlled conditions. When combined with ETS and EEG metrics, they allow for a nuanced understanding of how mental fatigue translates into tangible decreases in specific football actions, thereby increasing the applied value of the protocol for both research and practice.

The use of the ST as a method of inducing mental fatigue in this protocol is supported by a robust empirical basis that validates its effectiveness in overloading executive functions, namely inhibition and attentional control. The ST, by requiring constant resolution of conflicts between incongruent stimuli, intensely activates the anterior cingulate cortex, a region associated with conflict monitoring and cognitive effort (8). This sustained activation has been linked to reduced dopaminergic transmission, contributing to the subjective state of mental fatigue. Smith et al. (4) highlights that tasks such as the ST, due to their nature of inhibiting automatic responses, are particularly effective in inducing cognitive fatigue, making them suitable for experimental protocols that aim to simulate intense and prolonged mental demands.

In sporting contexts, Thompson et al. (5) observed that, despite its widespread use in studies on mental fatigue in football, the ST has limitations in terms of ecological validity, given that its repetitive and decontextualized structure differs from the complex cognitive demands experienced in real game situations. Nevertheless, its negative impact on physical and technical performance, as well as on decision-making, is well known, as evidenced by multiple studies. Marcora et al. (8), for example, demonstrated that after 90 min of a demanding cognitive task, participants showed a significant reduction in time to exhaustion during intense physical exercise, without relevant physiological changes, which reinforces the thesis that mental fatigue compromises physical performance mainly through an increase in the perception of effort. Thus, despite criticism regarding its contextual representativeness, the ST remains a reliable tool for experimental manipulation of mental fatigue, particularly in investigations that require strict control over the cognitive variables involved.

The statistical approach defined in this methodology and experimental protocol is structured to support the analysis of neurophysiological, visual, motor, and subjective outcomes using established quantitative methods. Descriptive statistics will characterize each variable, while paired and independent t-tests will assess intra- and intergroup differences before and after the intervention. A repeated measures ANOVA will be used to examine the effects of time and group interaction, with assumptions of normality and sphericity tested to ensure validity. Effect sizes (*η*^2^) and test power (π) will be reported to improve the interpretability and generalization of the results, following Marôco (90), Pallant (89), and Field (88). Pearson's correlation will be used to explore associations between domains (e.g., EEG activity and motor performance), interpreted according to Marôco (90). When appropriate, ANCOVA will control for individual variability, such as age or training experience. This analytical framework supports the protocol's goal of detecting the effects of mental fatigue on various dimensions of performance, based on robust methodological and statistical criteria.

### 3.1 Limitations and future perspectives

Despite the rigor of this protocol, there are some limitations to consider. The main one relates to the laboratory environment, which may not fully reflect the real playing conditions. Even with the use of football-specific tests (LSPT and Y-SART), it is not possible to completely reproduce the complexity, unpredictability and emotional charge of a competitive situation.

The present protocol is intentionally designed to investigate the acute effects of experimentally induced mental fatigue, as most existing experimental research in sport has focused on short-term cognitive manipulations [e.g., (1, 4, 8)]. Understanding these immediate neurophysiological and performance response is a necessary step before implementing longitudinal monitoring approaches. Future research should extend this framework to examine cumulative and seasonal effects of mental fatigue in applied football settings, and the current protocol provides a structured methodological basis for such longitudinal adaptations.

In the future, it will be useful to adapt this protocol to real training or game contexts, using mobile EEG and ETS. Longitudinal studies can show how repeated exposure to mental fatigue affects athletes' recovery and performance. It is also important to test strategies to reduce this fatigue, such as mental training, relaxation techniques, or individualized recovery plans. Finally, creating vulnerability profiles based on physiological and behavioral data can help prevent fatigue and improve performance in high-performance athletes.

## 4 Conclusion

This protocol proposes an innovative and integrated approach to studying the effects of mental fatigue on the motor performance of football players, combining subjective (VAS, FAS), neurophysiological (EEG), visual (ETS) and performance (LSPT, Y-SART) measures. By articulating these dimensions in the same quasi-experimental design, the aim is to capture more completely and realistically how mental fatigue manifests itself and compromises sports performance.

The main contribution of this study lies in its multimodal and sports-contextualized nature, offering a methodological model that can be replicated in research environments and applied to sports practice. This integration allows us not only to understand the immediate effects of fatigue, but also to advance in the recognition of patterns that may signal states of cognitive vulnerability in athletes.

From a practical point of view, the expected results can inform coaches, physical trainers, and sports psychologists about the importance of monitoring cognitive load in training. Early identification of signs of mental fatigue will allow for adjustments to training volume, the introduction of appropriate recovery strategies, and the personalization of interventions aimed at preserving athletes' decision-making capacity and motor efficiency. In the medium term, this methodology and experimental protocol may also serve as a basis for the development of monitoring technologies and systems that incorporate cognitive indicators into load control, contributing to performance optimization and the prevention of errors and injuries in high-performance contexts.

This protocol offers a rigorous and ecologically-grounded framework for examining how acute mental fatigue shapes neurophysiological activity, visual exploration, and football-specific performance. By integrating EEG, eye tracking, subjective ratings, and validated technical and agility tests within a single experimental structure; this protocol addresses key methodological gaps in the existing literature and provides a template that can be replicated and extended in different football settings.

The findings generated from this approach support more individualized monitoring of cognitive load, and inform training and recovery strategies; thereby helping practitioners better manage mental fatigue to optimize decision-making and technical execution in high-pressure football contexts.

## Data Availability

The raw data supporting the conclusions of this article will be made available by the authors, without undue reservation.
