# Correction: Mental fatigue in football: methodology and experimental protocol

**DOI:** 10.3389/fspor.2026.1901217

**Published:** 2026-07-02

**Authors:** Diogo Aveiro, Fernando Martins, Mário Pereira, Rodrigo Coimbra, Rodrigo José Mendes, João Pinto, John Kiely, Francisco Campos

**Affiliations:** 1 Department of Physical Education and Sport Sciences, University of Limerick, Limerick, Ireland; 2 Polytechnic University of Coimbra, Coimbra, Portugal; 3 SPRINT–Sport Physical activity and health Research & INnovation cenTer, Polytechnic University of Coimbra, Coimbra, Portugal; 4 Instituto de Telecomunicações—Delegação da Covilhã, Covilhã, Portugal; 5 iNED–Centre for Research & Innovation in Education, Polytechnic University of Coimbra, Coimbra, Portugal

**Keywords:** cognitive load, EEG, ETS, football, mental fatigue, motor performance

Affiliation 2, Polytechnic University of Coimbra, Coimbra, Portugal, was erroneously given as Department of Training of Educators and Teachers, Polytechnic University of Coimbra, Coimbra, Portugal.

Affiliation 2 was omitted for authors Mário Pereira, Rodrigo Coimbra, Rodrigo José Mendes, João Pinto, and Francisco Campos. This affiliation has now been added for these authors.

Affiliation 3, SPRINT—Sport Physical activity and health Research & INnovation cenTer, Polytechnic University of Coimbra, Coimbra, Portugal, was erroneously given as SPRINT—Sport Physical Activity and Health Research & Innovation Center, Polytechnic University of Coimbra, Coimbra, Portugal.

Authors Mário Pereira, Rodrigo Coimbra, Rodrigo José Mendes, João Pinto, and Francisco Campos were erroneously assigned to affiliation 6, Department of Education Sports and Social Intervention, Polytechnic University of Coimbra, Coimbra, Portugal. This affiliation has now been removed.

The original version of this article has been updated.

