# Correction: Mental fatigue in football: methodology and experimental protocol

**DOI:** 10.3389/fspor.2026.2003894

**Published:** 2026-10-13

**Authors:** Diogo Aveiro, Fernando Martins, Mário Pereira, Rodrigo Coimbra, Rodrigo José Mendes, João Pinto, John Kiely, Francisco Campos

**Affiliations:** 1 Department of Physical Education and Sport Sciences, University of Limerick, Limerick, Ireland; 2 Polytechnic University of Coimbra, Coimbra, Portugal; 3 SPRINT–Sport Physical activity and health Research & INnovation CenTer, Polytechnic University of Coimbra, Coimbra, Portugal; 4 Instituto de Telecomunicações—Delegação da Covilhã, Covilhã, Portugal; 5 iNED–Centre for Research & Innovation in Education, Polytechnic University of Coimbra, Coimbra, Portugal

**Keywords:** cognitive load, EEG, ETS, football, mental fatigue, motor performance

There was a mistake in **Figures 1**, **3** as published. The images corresponding to **Figures 1**, **3** were inadvertently interchanged. The corrected figures appear below.

The license information was missing from the caption of Figure 3. The corrected caption appears below.

**Figure 1:**
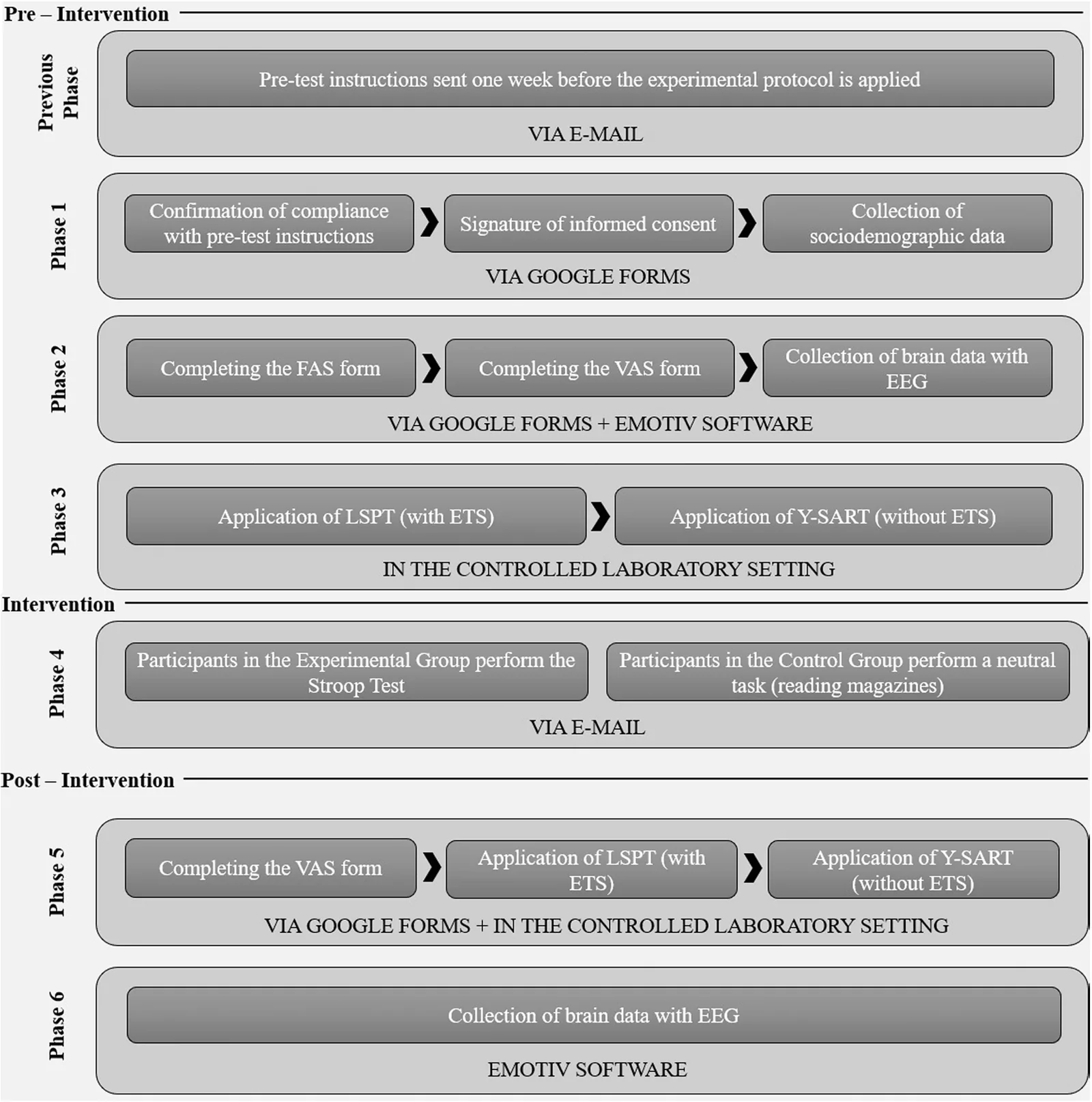
Study design.

**Figure 3:**
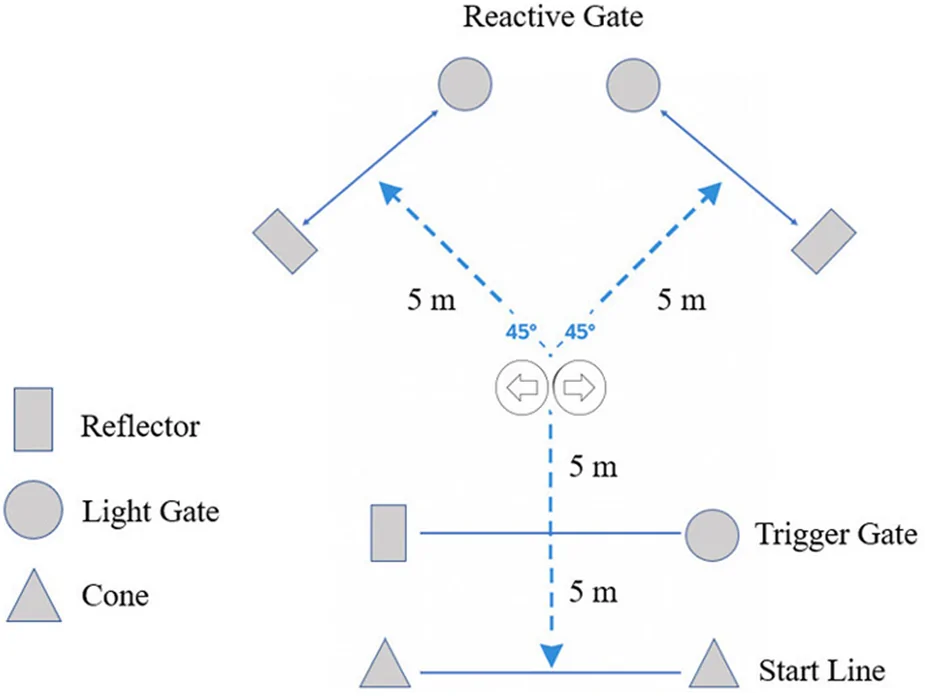
Schematic representation of the Y-SART, adapted from Yuan et al. (2), licensed under CC BY-NC-SA 4.0.

The original version of this article has been updated.

